# 

**Published:** 1877-09

**Authors:** 


The American Association for the cure of Inebriates will hold its Eighth Annual meeting at Chicago, Ill., Sept 12th, 1877. Important papers will be read and business transacted. President, T. L. Mason, M. D. Secretary, T. D. Crothers, M. D.

### Books and Pamphlets Received.

Ziemssen's Cyclopædia, Vol. XII. Diseases of the Brain and its Membranes. NewYork: Wm. Wood & Co., 1877. Buffalo: James H. Matteson.

Some general ideas concerning Medical Reform. By David Hunt, M. D. Boston: A. Williams & Co. New York: Wm. Wood & Co., 1877.

Report on the management of the Insane in Great Britain. By H. B. Wilbur, M. D. Albany: 1877.

Contributions to the treatment of Pulmonary Phthisis. By Dr. W. Gleitsmann.

A simple mode of cleansing the Nasal and Pharyngo—Nasal Passages. By Thomas F. Rumbold, M. D. St. Louis, Mo.

The Toner Lectures.—Lecture V.—On the Surgical complications and sequels of the continued Fevers. By Wm. W. Keen, of Philadelphia. Washington: Smithsonian Institute, April 1877.

Report upon Obstetrics. By Edward W. Jenks, M. D. Detroit, 1877.

Association of American Medical Colleges, History of its Organization. Detroit, 1877.

Transactions of the Medical Association of the State of Missouri. April 1877.

Popular Science Monthly, and Supplement for September, 1877.

Annual announcement of Indiana Medical College, 1877-8, and of Starling Medical College, 1877-8.

### Contents of September Number.

### ORIGINAL.

**Medical and Surgical Journal**

PUBLISHED MONTHLY,

Containing Original Articles, Reports of Medical Societies and Hospitals, Editorial, Reviews, Correspondence, Army News, etc., etc; including the usual variety of Medical Periodical Publications. Specimen copies sent on application.

Address Buffalo Medical & Surgical Journal, Buffalo, N. Y.

TERMS OF SUBSCRIPTION, $3.00 PER YEAR, IN ADVANCE.

